# Increased Hospitalizations for Neuropathies as Indicators of Zika Virus Infection, according to Health Information System Data, Brazil

**DOI:** 10.3201/eid2211.160901

**Published:** 2016-11

**Authors:** Christovam Barcellos, Diego Ricardo Xavier, Ana Luiza Pavão, Cristiano Siqueira Boccolini, Maria Fatima Pina, Marcel Pedroso, Dalia Romero, Anselmo Rocha Romão

**Affiliations:** Oswaldo Cruz Foundation, Rio de Janeiro, Brazil (C. Barcellos, D.R. Xavier, A.L. Pavão, C.S. Boccolini, M.F. Pina, M. Pedroso, D. Romero, A.R. Romão);; Instituto de Investigação e Inovação em Saúde–Universidade do Porto, Porto, Portugal (M.F. Pina)

**Keywords:** Zika virus, health surveillance, information systems, Brazil, neuropathies, Aedes

## Abstract

Evidence is increasing that Zika virus can cause extensive damage to the central nervous system, affecting both fetuses and adults. We sought to identify traces of possible clinical manifestations of nervous system diseases among the registers of hospital admissions recorded in the Brazilian Unified Health System. Time series of several diagnoses from the International Classification of Diseases, 10th Revision, were analyzed by using control diagrams, during January 2008–February 2016. Beginning in mid-2014, we observed an unprecedented and significant rise in the hospitalization rate for congenital malformations of the nervous system, Guillain-Barré syndrome, encephalitis, myelitis, and encephalomyelitis. These conditions are compatible with viral infection and inflammation-associated manifestations and may have been due to the entrance of Zika virus into Brazil. These findings show the necessity of adequately diagnosing and treating suspected cases of Zika virus infection and also that health surveillance systems can be improved by using routine data.

## Abstract 2

Neurologic manifestations of Zika infection must be adequately recognized and treated; our study methods can be used for monitoring and warning systems.

The recent spread of Zika virus across the globe has worried citizens and public health authorities. In late February 2016, the World Health Organization (WHO) declared an international state of emergency because of the advance of microcephaly associated with the Zika virus (*1*). Previously considered a minor infection with mild symptoms and a low mortality rate, infection with Zika virus was seen as less severe than that caused by other arboviruses transmitted by mosquitoes of the genus *Aedes*, such as dengue, yellow fever, and chikungunya viruses.

However, recent studies have shown that the infection has a high potential for causing damage to the central nervous system (CNS), leading to certain congenital malformations (*2*) and neuropathies, such as Guillain-Barré syndrome (GBS) (*3*–*6*). The neurotropic nature of the virus and its ability to cross the blood-brain and placental barriers (*7*) were demonstrated in laboratory experiments, and damage to developing brains has been shown by imaging of fetuses and newborns (*8*). Other neuropathies may be associated with the Zika virus and could have passed unnoticed through health information systems.

Some studies suggest that sporting events in 2013 and 2014 may have led to the introduction of Zika virus into Brazil (*9*). Further spread to other regions might have occurred by 2014. The first warnings of the epidemic in Brazil came on April 20, 2015 (*10*), with a report about the large number of cases of rash of unknown origin, which began at the end of December 2014 in various regions of the state of Pernambuco, followed by an alert issued by the state of Rio Grande do Norte (*11*). In May 2015, the spread of Zika virus among the local populations was laboratory confirmed, first in the Pernambuco and Bahia, then in other states of the center-west and southeast regions (*12*). The distribution of Zika virus in Brazil during 2014 and 2015 is summarized in Figure 1, according to case reports and epidemiologic data produced by the Federal Ministry of Health and state secretaries of health (*13*).

**Figure 1:**
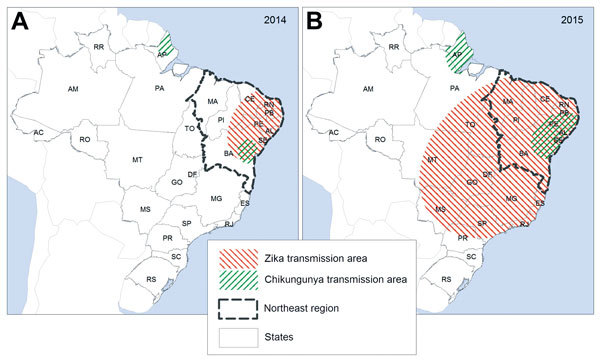
Approximate distribution of areas of local spread of Zika virus in Brazil, 2014 and 2015. Adapted from (*13*). State abbreviations: AC, Acre; AL, Alagoas; AP, Amapá; AM, Amazonas; BA, Bahia; CE, Ceará; GO, Goiás; DF, Distrito Federal; ES, Espírito Santo; MA, Maranhão; MT, Mato Grosso; MS, Mato Grosso do Sul; MG, Minas Gerais’ PA, Pará; PB, Paraíba; PR, Paraná; PE, Pernambuco; PI, Piauí; RJ, Rio de Janeiro; RN, Rio Grande do Norte; RS, Rio Grande do Sul; RO, Rondônia; RR, Roraima; SP, São Paulo; SC, Santa Catarina; SE, Sergipe; TO, Tocantins.

One strategy for acquiring morbidity data is to analyze hospital admission records. The Hospital Information System (SIH, in Portuguese) is primarily financial and administrative in nature, but it can be used as a data source for epidemiologic studies and surveillance because of advantageous characteristics, such as national coverage, relative consistency and standardization in terms of data generation, and rapid production and dissemination of data. These features facilitate its incorporation into epidemiologic alert systems, especially those for infectious diseases.

An analysis of hospital admissions data can provide a baseline that enables identification of trends in the number of admissions for neuropathies that affect the nervous system, as well as inflection points that depart abruptly from these trends. Long-term analyses of diseases potentially associated with Zika virus continue to be nonexistent in Brazil and have become urgently needed to bring into focus the scope of the problem, as well as delineate monitoring strategies for the epidemic in Brazil and other affected countries.

The main objective of this study was to analyze temporal patterns in hospitalizations in separate Brazilian regions during January 2008–February 2016, for neuropathies, which could have been associated with Zika virus infection in separate Brazilian regions. This period includes the time before the epidemic and the onset of the Zika epidemic in Brazil.

## Methods

This is a descriptive study of a time series from a population dataset. We sought to evaluate historical trends in neurologic conditions potentially related to infection with Zika virus, based on the principal diagnosis assigned at discharge as the main reason for hospitalization. Data were made available by the Informatics Department of the National Health System through the Hospital Information System during January 1, 2008–February 29, 2016, according to each patient’s state of residence.

We selected the health problems analyzed in this study on the basis of monitoring protocols put in place to track reports of microcephaly related to infection with Zika virus (*14*) and track neurologic conditions for which the patient had a history of previous viral infection (*15*). We considered all hospital admissions for the following: 1) congenital malformations of the nervous system (International Classification of Diseases, 10th Revision [ICD-10] Q00-Q07) in children <1 year of age, 2) GBS (ICD-10 G610; 3) other potential and unspecific clinical manifestations such as encephalitis, myelitis, and encephalomyelitis (ICD-10 G040-G049); 4) abortion and related problems (ICD-10 O03-O07); in particular, 5) spontaneous abortion (ICD-10 O03). In Brazil, abortion is permitted in cases of risk to the mother and fetus, as well as in cases of sexual violence. Other cases of induced abortion are attended to in hospitals when the woman initiates an abortion, when an incomplete abortion leads to complications, or in cases of hemorrhage or infection (*16*).

Time series were assembled by using aggregate data tabulated by month and region. Because the Northeast region was, until the end of 2015, the center of the Zika outbreak and of the surge in microcephaly cases, we analyzed this region and compared the results with those from the other Brazilian regions in aggregate. We calculated crude hospital admission rates for each month of the study using population estimates provided by the Brazilian Geography and Statistics Institute (Brazil). With respect to congenital malformations of the nervous system in children <1 year of age, we calculated the rate of hospitalizations using the number of live births per month as the denominator. In the case of abortion, we obtained the rate of hospitalizations using the number of live births per month as the denominator with a lag of 7 months, which reduced the effect of seasonality so common in data for births as well as for abortions.

The total number of hospitalizations can vary greatly by state due to variations in staffing at the local level and disparities in access to services for utilizers of the public health system (*17*). To control for the effect of the capacity of health services to provide hospital beds, we used the same techniques and parameters for the analysis of the general causes of hospitalizations, which include admissions for malformations among newborns (ICD-10, Chapter XVII: Congenital Malformations, Deformities and Chromosomal Anomalies) and all admissions for nervous system diseases (ICD-10, Chapter VI).

We compared the monthly hospitalization rates of the Northeast region with rates in non-Northeast Brazilian regions, added to a 95% CIs (1.96-fold SDs from the mean) as upper limits for detection of peaks in the time series. These data were plotted as control charts, which allowed for evaluation of long-term trends, seasonality, and anomalies in the series by visual inspection. We tested the difference between observed and expected number of hospitalization—assuming the historical baseline as a control parameter—by calculating the rate ratio (IRR) and CI using Poisson regression.

## Results

The rate of hospitalizations for congenital malformations of the nervous system (ICD-10 Q00-Q07) presented a stable mean value of 40/100,000 live births in the Northeast region until September 2015. As of November 2015, however, we observed an increase in this rate, which, in the last months of the series, reached a mean value of 170 hospitalizations for congenital malformations of the nervous system per 100,000 live births, an increase ≈4 times higher than historical rates (IRR 4.2; 95% CI 3.8–4.6). In February 2016, all regions exhibited rates of ≈100 hospitalizations for congenital malformations of the nervous system per 100,000 live births, a 2-fold increase over the national historical baseline. From November 2015 through February 2016, a total of 1,027 hospitalizations for congenital malformations of the nervous system were recorded nationwide; 448 of these occurred in the Northeast region. This region is responsible for ≈830,000 (28%) of the overall annual live births in Brazil.

In the Northeast region, the hospitalization rate for GBS (ICD-10 G610) was 0.05/100,000 residents until May 2015, when an outbreak occurred, which peaked in July 2015. From June 2015 through February 2016, the hospitalization rate was 0.11/100,000 residents, an increase by a factor of 2.7 (95% CI 2.5–3.0). During July through October 2015, 377 GBS hospitalizations were recorded in the Northeast region, an excess of ≈240 hospitalizations in the region.

The hospitalization rate for CNS inflammatory diseases, represented by encephalitis, myelitis, and encephalomyelitis (ICD-10 G040-G049), increased in the Northeast region, principally as of September 2014, from a baseline of 0.05 hospitalizations/100 residents to 0.11 hospitalizations/100,000 residents (IRR 2.0; 95% CI 1.9–2.2). In the rest of the country, a stable base line of 0.05 hospitalizations/100,000 residents was observed. From September 2014 through February 2016, hospitalizations for encephalitis, myelitis, and encephalomyelitis in the Northeast region reached 1,115, an excess of ≈570 hospitalizations.

**Figure 2:**
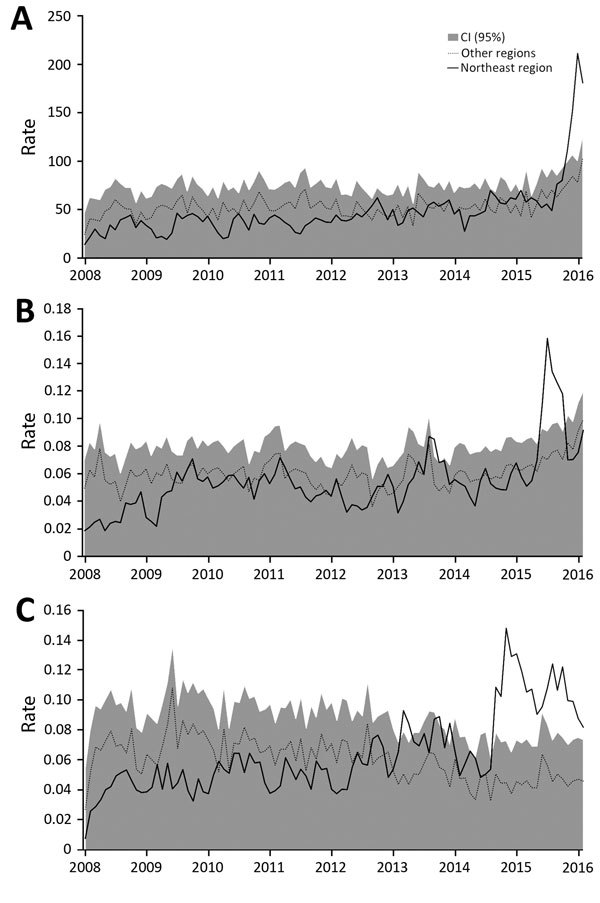
Hospitalizations in the Northeast region of Brazil, indicated by codes from the International Classification of Diseases, 10th Revision, January 2008 through February 2016. A) Congenital malformations of the nervous system (ICD-10; Q00-Q07) per 100,000 live births; B) Guillain-Barré syndrome (ICD-10; G610) per 100,000 residents; and C) encephalitis, myelitis and encephalomyelitis (ICD-10; G040-G049) per 100,000 Northeast region residents, compared with hospitalizations for these conditions in the rest of the country. Source: Hospital Information System (SIH); www.datasus.gov.br.

Figure 2 shows the evolution during 2008–2016 of the rate of congenital malformations of the nervous system in children <1 year of age (per 100,000 live births) and of the rates of hospitalization (per 100,000 residents) for GBS and encephalitis, myelitis, and encephalomyelitis in the Northeast region compared with rates in the rest of the country. Figure 2, panel A, shows the abrupt increase in hospitalization rates for congenital malformations of the nervous system among children <1 year of age as of October 2015 in the Northeast region. Meanwhile, the rest of the country exhibited a slight increase in these same rates. Some peaks of hospitalizations can be seen in August 2014 and February 2015, when the rate observed in the Northeast region surpasses the CI for the rate in the rest of the country.

With respect to GBS (Figure 2, panel B), hospitalizations peaked sharply during June–August 2015 in the Northeast region. In a more detailed analysis, we identified the first peak of GBS in Pernambuco State during May 2015, followed by the states of Ceará, Bahia, and Alagoas in the subsequent months. This trajectory is consistent with the early reports of Zika and rash epidemic epidemiologic alerts (*11*,*12*).

A historical series of the rates of hospitalization for inflammatory diseases of the CNS, represented by encephalitis, myelitis, and encephalomyelitis, shows a pattern change in recent years (Figure 2, panel C). Until 2013, the rates of the series in the Northeast region varied within the range of the rest of Brazil. By the end of 2014, the Northeast region showed an increase in relation to the rest of the country, with values above the CIs for the series for the rest of Brazil. In mid-2015, the number of hospitalizations peaks again during August–October. This pattern suggests a more accentuated annual cycle, showing peaks during the second semester of the 2014 and 2015.

We did not observe changes for abortions and related problems in the times series, including for spontaneous abortions (results not shown). We also did not observe changes in the total number of hospitalizations or in hospitalizations for nervous system problems (ICD-10, Chapter VI) in the regions during the study period. This pattern may demonstrate the stability of the health system in terms of specialty beds, especially for neurology.

## Discussion

In this study, we analyzed time trends for hospitalizations for some neuropathies. We found increases in the number of hospitalizations for congenital malformations of the nervous system, GBS, and some inflammatory diseases of the CNS. These complications began to cause more hospitalizations, with strong fluctuations over the course of the study period, beginning even before the first warning in November 2015 about the possible effects of infection with Zika virus on microcephaly and other malformations. These shifts from the previous baseline were not, therefore, influenced by new procedures and norms that have been incorporated into the health system over the last months. As such, the complications that we present here can be thought of as early signals of neurologic complications that were unperceived through health surveillance systems, but that may be related to the entrance and circulation of the Zika virus in Brazil, especially in the Northeast region.

Our results show that the rates of hospitalization in the Northeast region for some neuropathies were subject to abrupt changes beginning in mid-2014, when it is believed that Zika virus was introduced to the country (*9*) (and later produced outbreaks in 2015). Conversely, the onset of hospitalizations for congenital malformations of the nervous system observed in November 2015 may be a result of the first public health warnings about Zika-related microcephaly, issued in the same month. Taking into consideration the fact that CNS malformations originate, typically, in the first 3 months of gestation (*18*), infection may have occurred in mid-2015, which coincides with the observed peaks of GBS and CNS inflammatory diseases. The hospitalization rates have slightly increased nationally during the first months of 2016, probably in reaction to warnings about microcephaly and the ensuing necessity to keep neonates hospitalized to investigate potential cases.

With respect to problems related to abortion, including spontaneous abortion, hospitalizations rates remained stable over the study period. We expected an increase in the number of abortions and related problems, especially spontaneous abortions, which can occur in cases of serious congenital abnormalities (*19*). Equally, public health warnings about the surge in microcephaly cases may have provoked an increase in the number of induced abortions. In Brazil, legislation permits abortion in cases of risk to the mother and fetus, or in cases of sexual violence, but studies have shown that health information systems substantially underestimate abortion, and also document bureaucratic delays of health systems in performing legal abortions (*16*). The possibility of an increase in the number abortions and related complications in the coming months should not be discounted.

The concentration of neuropathies in the Northeast states remains a mystery for researchers and health surveillance services. First, we can assume that the virus entered Brazil mainly in this region (*12*,*20*) and that, with the spread of the epidemic to other regions, the number of cases of neuropathies will increase in the rest of the country. On the other hand, the theory persists that other risk factors, such as chronic diseases (*21*) and co-infections (*22**,**23*) might interact with and potentiate neurologic manifestations in individuals infected by the Zika virus. The region had one of the worst droughts in its history during 2010–2013, resulting in contamination of drinking water, food insecurity (*24*), and outbreaks of diarrhea across the region (*25*), which could have affected the immunity status of the population. In addition, concomitant outbreaks of Zika virus infection and dengue, as was observed in French Polynesia (*26*) and in northeast region of Brazil, could potentiate the neurologic effects of infection with Zika virus. Chikungunya virus was also circulating in the region during 2014 and 2015, although in more restricted areas (Figure 1) and with less magnitude, and it can also produce severe neurologic complications such as GBS (*27*).

Another issue is the higher rate of hospitalizations during the winter months (June–September), when in general the incidence of vector-transmitted diseases, such as dengue, is lower because of milder temperatures (*28*). In this case, we can presume a latency period between infection with the Zika virus and hospitalization for neurologic complications. A study carried out in Salvador, a chief city in the Northeast region, demonstrated a lag of 5–9 weeks (mean 2 months) between the Zika virus epidemic and cases of GBS (*29*). Another hypothesis is that transmission of the virus persists beyond seasonal limits, due to other means of transmission, such as through sexual contact (*30*).

Our study has certain limitations. First, a detection bias could exist (i.e., the alerts about the Zika epidemic could have led to greater rigor on the part of health services to diagnose and register the neuropathies and congenital abnormalities we have examined, which would result in overestimation of hospitalization rates). However, because the first alerts about microcephaly related to Zika virus infections were issued in November 2015, and only in February 2016 did WHO declare a public health emergency, the previous months’ records may have not been affected by health surveillance guidelines, with the exception of microcephaly.

A second limitation concerns the fact that the health information system for hospitalizations registers events and not individual patients. As such, rehospitalizations can be contained within that figure, leading to overestimation of hospitalization rates. These 2 limitations likely do not substantially affect the results of this study because they would have had to occur uniformly across the nation in the period studied.

This study can contribute to a greater understanding of the Zika epidemic in Brazil and its additional effect on certain neuropathies. The data sources and methods we used in this study can be used for monitoring and warning systems in cases of emergency. Implementation would require obtaining and statistically analyzing time series data, to establish a baseline of adverse events and identify peaks that could represent outbreaks of disease.

To monitor the effects of Zika virus, public health researchers must consider its various clinical manifestations that may be registered in different ICD codes, even in dispersed chapters, such as viral encephalitis (ICD-10 A83) and provisional code (ICD-10 U06), as recommended by WHO. The hospital routine may account for the diversity of codes used to describe complications derived from Zika virus. The admission of a patient to the hospital is first justified by a preliminary ICD code. Throughout the period of hospitalization, new examinations and procedures are carried out, which can alter this classification. At the end of hospitalization period, a code for the principal diagnosis is assigned to the outpatient, based on procedures performed and their costs, which is not necessarily related to the disease etiology. A wide range of ICD codes should be taken into account in this situation, with anticipation of all possible reasons for hospital admission potentially related to Zika complications.

New detection tools for outbreaks and means of communicating warnings should be pursued to identify real trends and, at the same time, minimize false alarms and panic that could be provoked in populations potentially affected by the Zika epidemic. The history of neuropathies caused by viruses is about to change, not only because of the effects that Zika virus has been found to have on the CNS, but also because of the effort of gathering evidence that will help construct this history.
